# Risk of repeat self-harm among individuals presenting to healthcare services: development and validation of a clinical risk assessment model (OxSET)

**DOI:** 10.1136/bmjment-2024-301180

**Published:** 2024-10-15

**Authors:** Seena Fazel, Maria D L A Vazquez-Montes, Tyra Lagerberg, Yasmina Molero, Jane Walker, Michael Sharpe, Henrik Larsson, Bo Runeson, Paul Lichtenstein, Thomas R Fanshawe

**Affiliations:** 1 Department of Psychiatry, Warneford Hospital, University of Oxford, Oxford, UK; 2 Oxford Health NHS Foundation Trust, Oxford, UK; 3 Nuffield Department of Primary Health Care Sciences, University of Oxford, Oxford, UK; 4 Department of Medical Epidemiology and Biostatistics, Karolinska Institutet, Stockholm, Sweden; 5 Department of Clinical Neuroscience, Karolinska Institute, Stockholm, Sweden; 6 School of Medical Sciences, Örebro University, Örebro, Sweden

**Keywords:** Suicide & self-harm, Data Interpretation, Statistical

## Abstract

**Background:**

A self-harm episode is a major risk factor for repeat self-harm. Existing tools to assess and predict repeat self-harm have major methodological limitations, and few are externally validated.

**Objective:**

To develop and validate a risk assessment model of repeat self-harm up to 6 months after an episode of non-fatal self-harm that resulted in an emergency visit to hospital or specialised care.

**Methods:**

Using Swedish national registers, we identified 53 172 people aged≥10 years who self-harmed during 2008–2012. We allocated 37 523 individuals to development (2820 or 7.5% repeat self-harm incidents within 6 months) and 15 649 to geographic validation (1373 repeat episodes) samples, based on region of residence. In a temporal validation of people who self-harmed during 2018–2019, we identified 25 036 individuals (2886 repeat episodes). We fitted a multivariable accelerated failure time model to predict risk of repeat self-harm.

**Findings:**

In the external validations (n=40 685), rates of repeat self-harm were 8.8%–11.5% over 6 months. The final model retained 17 factors. Calibration and discrimination were similar in both validation samples, with observed-to-expected ratio=1.15 (95% CI=1.09 to 1.21) and c-statistic=0.72 (95% CI=0.70 to 0.73) in the geographical validation. At 6 months and a 10% risk cut-off, sensitivity was 51.5% (95% CI=48.8% to 54.2%) and specificity was 80.7% (95% CI=80.1% to 81.4%) in geographic validation; corresponding values were 56.9% (95% CI=55.1% to 58.7%) and 76.0% (95% CI=75.5% to 76.6%) in temporal validation. Discrimination was slightly worse at the 1-month prediction horizon (c-statistics of 0.66–0.68).

**Conclusions:**

Using mostly routinely collected data, simple risk assessment models and tools can provide acceptable levels of accuracy for repeat of self-harm.

**Clinical implications:**

This risk model (OXford SElf-harm repeat tool) may assist clinical decision-making.

**WHAT IS ALREADY KNOWN ON THIS TOPIC**

- Individuals who self-harm are at high risk of repeat self-harm episodes—tools that accurately assess and model repeat self-harm would be valuable to support clinical decision-making. However, existing instruments suffer from major methodological flaws, limiting their applicability.

**WHAT THIS STUDY ADDS**

- We have developed and extensively validated a simple and transparent assessment tool that draws on routinely collected information, with acceptable performance.

**HOW THIS STUDY MIGHT AFFECT RESEARCH, PRACTICE OR POLICY**

- This new tool can contribute to transparent and evidence-based decision-making regarding safety planning, follow-up and addressing psychosocial needs for individuals presenting to emergency care with self-harm.

## Background

 Self-harm is the third largest contributor to disability-adjusted life years among those aged 10–24 years globally, and the 22nd largest across all ages—with 16 million people engaging in self-harm each year worldwide.^1^ Prevention is key for an outcome that causes significant harm—to the affected individual, their carers and families and society at large.

Risk stratification through accurate risk assessment models is one approach to improve the prevention of adverse outcomes by targeting resources towards those at highest risk, to reduce the inconsistency and variability in how risk is assessed within and between clinical services, and mitigate against cognitive biases (including recency, optimism and confirmation bias) that influence clinical decision-making. Such models can also increase transparency in these assessments, which can in turn improve how risk is communicated to those asssessed and their carers. A particularly high-risk group are those who have previously engaged in self-harm.^2^ However, few assessment models and tools estimate the risk of future self-harm in this population. Existing models have significant limitations, including small development samples,^3 4^ random splitting of training (development) and testing (validation) datasets,^5^ outdated methods^6 7^ and stratifying individuals based on a risk factor checklist without any statistical modelling.^8^

We have previously developed the OxSATS prediction tool to estimate the risk of suicide mortality after a self-harm presentation, with good predictive performance.^9^ While many risk factors for suicide and self-harm are overlapping, and there are theoretical justifications for predicting both self-harm and suicide in one model, non-fatal self-harm has distinct predictors. For example, one of the strongest risk factors for self-harm—sex at birth—has a different direction of association than it does with suicide mortality, with females having a higher risk of repeat self-harm than males.^10^ In addition, another important predictor is age, and the age structure is different for repeat self-harm than suicide.

## Objective

The aim of this study is to develop a risk assessment tool for repeat non-fatal self-harm. To comprehensively assess clinical applicability of the model, we include both a geographic and temporal external validation, as well as a validation using a composite outcome of repeat non-fatal self-harm and death by suicide.

## Methods

### Study design

This cohort study used data from the following total population Swedish registers: Total Population Register, Multi-Generation Register, Patient Register (healthcare episodes in secondary care in the public system), Cause of Death Register, Longitudinal Integrated Database for Health Insurance and Labour Market Studies, Prescribed Drug Register, National Crime Register and Register of Persons Suspected of Offences.^11^ The index population was defined as individuals aged at least 10 years (as it was assumed those under 10 had their diagnosis misclassified), who made an emergency visit to hospital or specialist (psychiatric and other) healthcare for non-fatal self-harm (ICD-10 codes X60–X84 and Y10–Y33, but excluding Y34: method of self-harm not recorded) between 1 January 2008 and 31 December 2012. Individuals referred directly to psychiatric care or who died during their hospitalisation for self-harm were excluded. Some individuals had more than one self-harm visit during the study period, and in these cases a single randomly selected visit was chosen as the index self-harm event. This approach is representative of the intended use of the model which is to predict the risk of repeat self-harm in new individuals using information on past self-harm.

For a temporal external validation, we included any self-harm by the above definition occurring between 1 January 2018 until 31 December 2019. All other selection criteria were the same.

### Outcome definitions

The follow-up period was until 31 December 2013; for the temporal validation sample it was up to 30 June 2020. The repeat self-harm outcome was defined as the occurrence of a second, subsequent self-harm event after the index event of an emergency visit to hospital or specialist healthcare for self-harm behaviour with ICD-10 codes X60–X84 or Y10–Y33, excluding Y34. For individuals with more than one subsequent self-harm event, the time of the first such event was used. The risk tool aimed to assess the probability of repeated self-harm within two prediction horizons: (1) within 1 month and (2) within 6 months of the index self-harm event. Repeat self-harm events were counted only if the admission date fell at least 1 day after the date of discharge of the index event, to avoid double counting the index event when an individual is transferred to other units within or between hospitals. In a validation using a composite outcome of repeat self-harm and suicide death, we included deaths where the cause of death was listed as ICD-10 codes X60–X84 or Y10–Y33, excluding Y34.

### Risk factors

We predefined a set of 33 risk factors for inclusion in the assessment tool. These included a range of variables for which previous studies have indicated a possible association with repeated self-harm (online supplemental table 1).^4^,^812^ For time-varying risk factors, assessment at the time of the index event was used. We assigned risk factors into two groups: the first contained variables that would be included in the statistical model irrespective of statistical significance (age, sex, alcohol and drug use disorder, lifetime and previous 12-month history of self-harm), based on evidence of their association with repeat self-harm in the research literature; the second group contained risk factors to be selected using backward stepwise selection (5% significance level), thus adjusting for overfitting. Interactions between predictors were not considered as additional predictors, with the exception of two predefined interactions (age×sex and age×lifetime history of self-harm).^19^

### Statistical methods

Given that the proportional hazards assumption was not satisfied for the age variable, we fitted a multivariable accelerated failure time model with Weibull errors to investigate the associations between risk factors and the time to first repeat self-harm event. Model fitting used the full period of follow-up available for each individual, with censoring at the administrative end of follow-up or death from any cause. Fractional polynomials were used to model the effect of the age covariate (in years) to account for non-linear effects. The final model is presented after accounting for optimism (the tendency of predictive models to perform better in development samples than external populations) by multiplying every estimated model coefficient by the uniform shrinkage factor calculated as (the model’s χ^2^–df)/model’s χ^2^).^20^ Living situation was the only factor with missing data (2% in the development sample; 1% in the geographic validation sample). Multiple imputation was not performed for missing data.

Predicted probabilities of self-harm within prediction horizons of 1 and 6 months after index self-harm were obtained using the final model. Internal discrimination assessment, using Harrell’s c-statistic,^21^ was performed using bootstrapping^20^ (subtracting the average difference between discrimination measures from bootstrap created models evaluated in their bootstrap samples from uncorrected discrimination of the original model) to correct for optimism, that is, the tendency of predictive models to perform better in development than external samples. The Harrell’s c-statistic gives a measure of the proportion of observations that the model is able to correctly order in terms of their survival time—that is, the better the discrimination, the more likely the model is to assign a higher risk to a person with the event than to a person without the event.^21^ We also calculated an optimism-corrected Somers’ D statistic (non-parametric measure of strength and direction of association between ordinal dependent and independent variables) and area under the receiver operating characteristic (ROC) curve (AUC). We produced calibration plots to compare predicted and observed probabilities of repeated self-harm, estimated calibration slope and intercept and calculated ratios of observed to expected (O:E) number of events. A calibration slope of 1 and intercept of 0 would indicate perfect correspondence between observed and predicted probabilities and therefore perfect calibration, as would an O:E of 1.

To allow for comparability and enhance usability, we report additional performance measures (sensitivity, specificity, positive and negative predictive values) at two predetermined cut-points (10% and 20%) based on anticipated outcome rates and interpretability. 95% CIs are presented where appropriate. The model was implemented into an online risk calculator, called OXford SElf-harm repeat Tool (OxSET, available at https://oxrisk.com/oxset/). We extracted the data using SAS software V.9.4 and analysed it using StataSE V.16 (StataCorp).

Transparent Reporting of a multivariable prediction model for Individual Prognosis or Diagnosis (TRIPOD) guidelines were followed for the design and reporting of this work.^22^

### Validation

For the geographic validation, we used the residential location of the individual at the time of admission or the year before or after, if missing, as a marker of geographical location. See online supplemental text for selection of geographic regions. For the temporal validations, we only extracted risk factors included in the final prediction model.

There were a total of 648 first-time repeat self-harm episodes within 1 month after an index self-harm event, and 1373 within 6 months in the geographic validation sample; the corresponding figures were 1591 and 2886 for the temporal validation sample. In a separate analysis using the composite outcome (self-harm and suicide) in the temporal validation sample, the corresponding number of events was 1702 and 3101. This far exceeds the minimum recommended events for external validation studies.^23^

### Findings

Of the 53 172 people who self-harmed in the study cohort, 37 523 were assigned to model development and 15 649 to the geographic external validation sample; a further 25 036 individuals were identified for temporal external validation. In the development sample, numbers who experienced a repeat self-harm event within 1 month and 6 months of follow-up were 1259 (3.4%) and 2820 (7.5%), respectively. The repeat self-harm rate was higher in the geographic and temporal validation samples (online supplemental table 2). Online supplemental table 3 shows the event rates for repeated self-harm in the regions forming each of the development and geographic validation sample.

From the set of predetermined risk factors and further risk factors selected using backward stepwise selection, we retained 17 risk factors in the final model. Table 1 summarises the distribution of these 17 risk factors in the development and validation samples. In the development sample, individuals had a median age of 32 (IQR 21–49) years, 55% were women, and many received any psychotropic (56%) or antidepressant medication (33%) in the 3 months prior to the self-harm index date. 30% of individuals had a lifetime history of self-harm and 44% had a psychiatric disorder diagnosis in the previous 12 months. 40% had a family history of any psychiatric disorder. The risk factors in the geographic external validation sample had a similar distribution to those in the development sample. Online supplemental table 4 summarises further characteristics of the development and geographic external validation sample—all reported characteristics were considered as candidate risk factors in the model. The temporal validation sample had some differences suggestive of a higher risk population (table 1), which included a higher proportion of individuals with drug use disorders (31% vs 22% in the development sample), lifetime history of self-harm (37% vs 30%) and family history of psychiatric disorders (60% vs 40%).

**Table 1:** Distribution of the risk factors for repeat self-harm included in the final multivariable model

	Development sample N=37 523		Geographic validation sample N=15 649		Temporal validation sample N=25 036	
General demographics	N	%	N	%	N	%
Age (years)*	Median=32, IQR=21–49		Median=33, IQR=21–49		Median=31, IQR=21–50	
Sex, female*	20 561	54.8	8685	55.5	13 839	55.3
Substance misuse						
Current or lifetime alcohol use disorder*	7257	19.3	3025	19.3	4979	19.9
Current or lifetime drug use disorder*	8384	22.3	3672	23.5	7657	30.6
Living situation						
Living with other adult	14 151	37.7	5697	36.4	7958	31.8
Treatment in the past 3 months						
Any psychotropic medication	20 888	55.7	9537	60.9	11 212	44.8
Antidepressant treatment	12 527	33.4	6009	38.4	9636	38.5
Antipsychotic treatment	4259	11.4	1834	11.7	4032	16.1
Mood stabiliser treatment	677	1.8	310	2.0	1714	6.9
History of self-harm						
Method of index self-harm event† Cutting Hanging, strangulation or suffocation	4925 308	13.1 0.8	1614 150	10.3 1.0	4620 565	18.5 2.3
Lifetime history of self-harm prior to index*	11 277	30.1	5372	34.3	9140	36.5
History of self-harm in the 12 months prior to index*	4281	11.4	1994	12.7	4022	16.1
Number of lifetime prior self-harm episodes 1–2 episodes 3+ episodes	31 740 5783	84.6 15.4	12 879 2770	82.30 17.7	19 707 5329	78.71 21.3
Time between episodes ≤1 month	1935	5.2	838	5.4	1943	7.8
Mental health in the past 12 months						
Any psychiatric disorder (except substance use disorders)	16 472	43.9	7281	46.5	13 140	52.5
Family history						
Family history of any psychiatric disorder	15 112	40.3	6503	41.6	15 012	60.0

Values are all numbers, percentages, or 95% CIs, except for age for which mean and SD are reported.

* Core factor, kept in the final model independently of its statistical significance or predictive strength.

† It was possible for an individual’s index self-harm event to be coded under more than one method. See online supplemental table 1 for definitions of self-harm and other predictors.

The median follow-up time in the development sample was 37 months (IQR 20–55). Among the 5813 individuals who had repeat self-harm at any time during follow-up, the median event-free follow-up was 6 months (IQR 1–18). The remaining 31 710 censored individuals (ie, those who did not self-harm within 6 months) had a median follow-up of 42 months (IQR 26–57). Similar follow-up times were observed in the geographic external validation sample.

In the fitted model of 17 risk factors (table 2), factors associated with higher risk of repeat self-harm were younger age, female sex, current or lifetime alcohol and drug use disorders, living alone, dispensation of psychotropic medication (including antidepressant, antipsychotic or mood stabiliser) within 3 months prior to index self-harm, lifetime and recent history of self-harm, recent and family history of psychiatric disorder, and an index self-harm event attributed to cutting, hanging, strangulation or suffocation. The interaction of age and sex was retained, with young females being at higher risk of repeat self-harm than young males, and a sharper rate of decline in risk with age for females than for males (online supplemental figure 1). The full model prediction equation is presented in online supplemental table 5. No polynomial transformation was needed for age. Unadjusted associations between candidate predictors and outcome are shown in online supplemental table 6.

**Table 2:** Risk factors (with HRs and 95% CIs) in the final multivariable model

	HR	95% CI		P value
General demographics				
Age in decades	0.92	0.90	0.95	<0.001
Female sex	1.65	1.41	1.92	<0.001
Interaction of age (in decades) and sex	0.92	0.88	0.95	<0.001
Substance misuse				
Current or lifetime alcohol use disorder	1.27	1.18	1.36	<0.001
Current or lifetime drug use disorder	1.45	1.35	1.55	<0.001
Living situation				
Living with other adult	0.92	0.86	0.98	0.011
Treatment in the past 3 months				
Any psychotropic medication	1.47	1.35	1.62	<0.001
Antidepressant treatment	1.17	1.09	1.26	<0.001
Antipsychotic treatment	1.20	1.11	1.29	<0.001
Mood stabiliser treatment	1.19	1.02	1.38	0.024
History of self-harm				
Method of index self-harm event Cutting Hanging, strangulation or suffocation	1.31 1.63	1.20 1.27	1.42 2.10	<0.001 <0.001
Lifetime history of self-harm prior to index	1.23	1.12	1.34	<0.001
History of self-harm in the 12 months prior to index	1.73	1.58	1.89	<0.001
Number of lifetime prior self-harm episodes, 3+ episodes	1.71	1.57	1.86	<0.001
Any self-harm occurring within 1 month prior to index date	1.12	1.01	1.24	0.038
Mental health in the past 12 months				
Any psychiatric disorder (except substance use disorders)	1.45	1.35	1.56	<0.001
Family history				
Family history of any psychiatric disorder	1.10	1.03	1.16	0.002

### Internal performance

The optimism-corrected Harrell’s c-statistic for the overall internal discrimination performance was 0.73 (95% CI 0.73 to 0.74), and Somer’s D statistic was 0.47 (95% CI=0.45 to 0.48) (online supplemental table 7). Online supplemental figure 2 presents the ROC curves and corresponding AUCs when using the model to predict risk of repeat self-harm within 1 and 6 months in the development sample. The median predicted risk within 1 month was 2% (IQR 1%–4%), within 6 months it was 5% (IQR 3%–8%) and the predicted risk was lower than the prespecified cut-point of 25% for 98% of individuals within 1 month and 94% within 6 months. Of the two alternative cut-points explored (10% and 20%), 10% had the highest sensitivity while maintaining specificity above 80% (online supplemental figure 2, online supplemental table 8). Online supplemental figure 3 presents the calibration plots, intercepts, slopes and O:E ratios for risk prediction within 1 and 6 months in the development sample. The model showed good internal calibration, slightly underestimating large risk scores at 1 month and overestimating them at 6 months.

### External performance

In geographic external validation, c-statistics for the 1-month and 6-month prediction horizons were 0.68 (95% CI=0.65 to 0.70) and 0.72 (95% CI=0.70 to 0.73), respectively. The corresponding Somers’ D statistics were 0.35 (95% CI=0.31 to 0.40) and 0.43 (95% CI=0.40 to 0.46).

In the temporal external validation sample, the c-statistic was 0.66 (95% CI=0.64 to 0.67) for the 1-month horizon and 0.70 (95% CI=0.69 to 0.71) for 6-month follow-up. D values were 0.31 (95% CI=0.28 to 0.34) and 0.41 (95% CI=0.39 to 0.43), respectively. Harrell’s c and Somer’s D statistics in the temporal validation sample when using a composite outcome of self-harm and suicide were almost identical to those where only repeat non-fatal self-harm was considered (online supplemental table 9).

**Figure 1:**
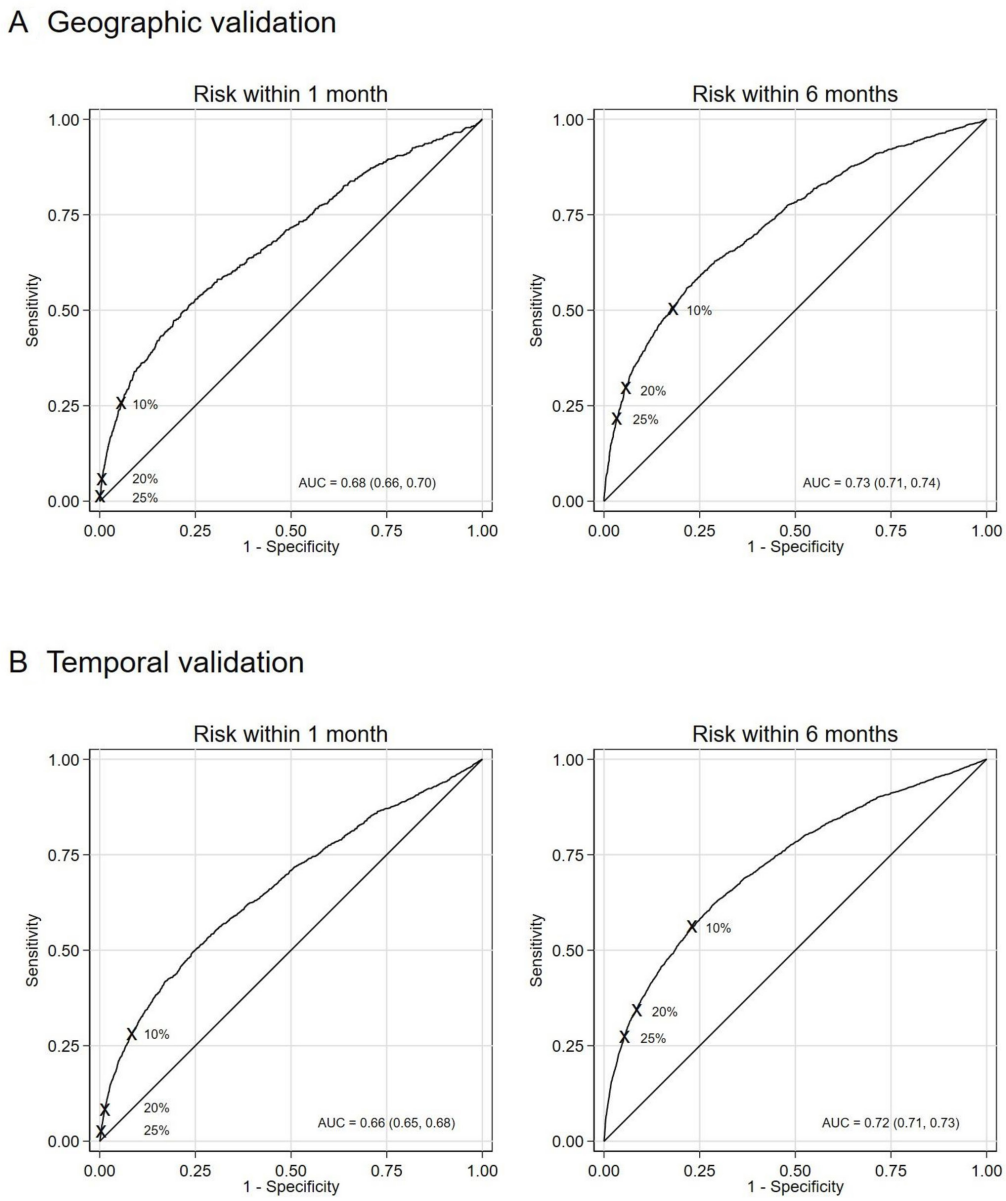
Receiver operating characteristic (ROC) curve and area under the ROC curve (AUC) (95% CI) for the final model when used to predict risk of repeat self-harm at 1 and 6 months in the external validation samples. Sensitivity and specificity for the risk thresholds considered (10%, 20% and 25%) are shown.

Figure 1 presents the ROC curves and corresponding AUCs when using the model to predict risk of repeat self-harm within 1 and 6 months in the external validation samples, and online supplemental table 10 provides further details of the classification measures, including positive and negative predictive values. Classification measures for the external validations were similar to those obtained in the development sample. In the geographic external validation at the 10% cut-point, the model’s sensitivity and specificity to predict risk of repeat self-harm within 1 month were 26.4% (95% CI=23.0% to 30.0%) and 93.1% (95% CI=92.7% to 93.5%), respectively; and within 6 months 51.5% (95% CI=48.8% to 54.2%) and 80.7% (95% CI=80.1% to 81.4%), respectively. For the temporal external validation, corresponding sensitivity and specificity were 28.6% (95% CI=26.4% to 30.9%) and 91.3% (95% CI=91.0% to 91.7%) within 1 month; and 56.9% (95% CI=55.1% to 58.7%) and 76.0% (95% CI=75.5% to 76.6%) within 6 months. These figures were similar when using the composite outcome in the temporal validation sample (online supplemental figure 4).

**Figure 2:**
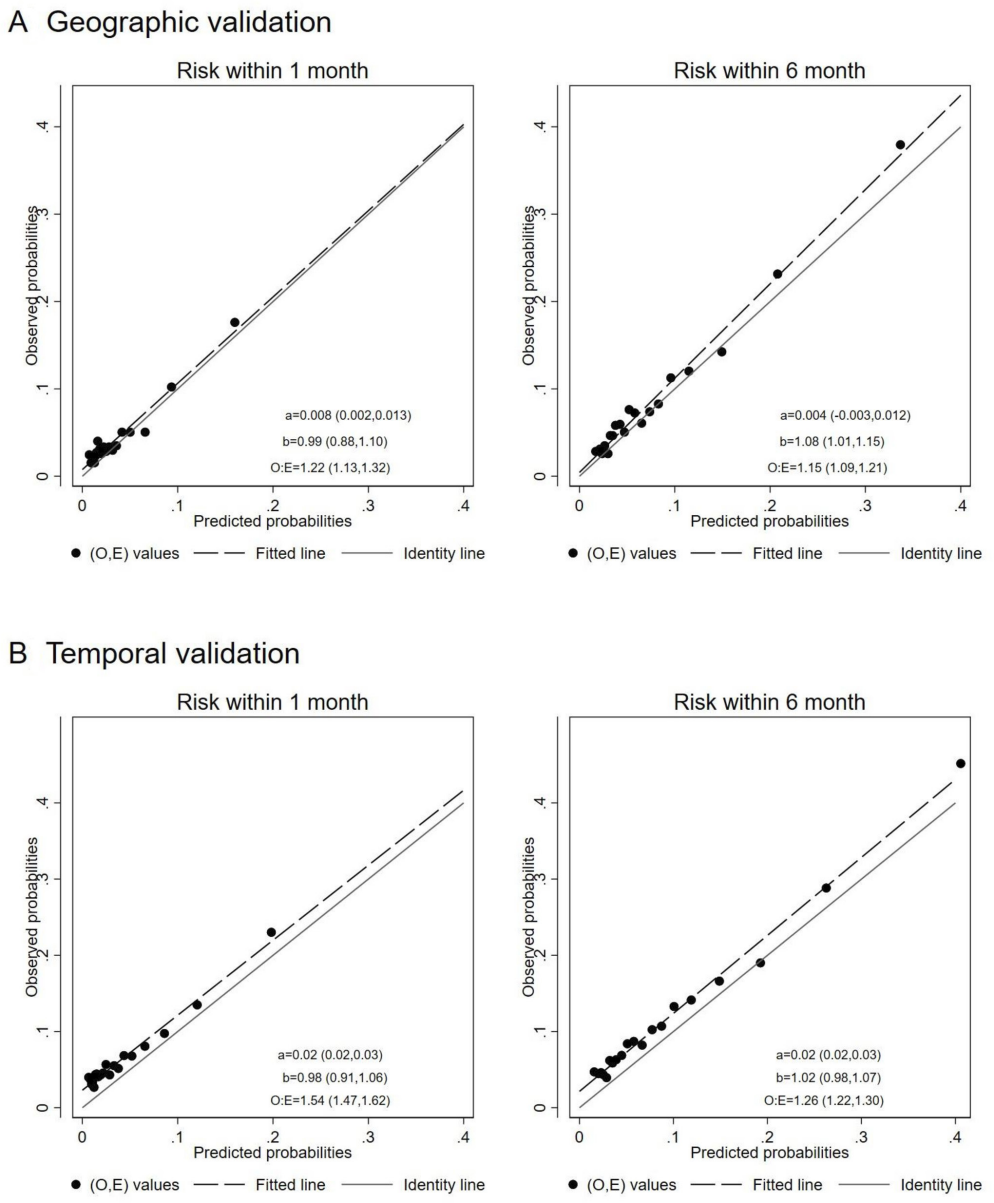
Calibration plots for the final multivariable model used to predict repeat self-harm in the external validation samples. a, b, calibration intercept and slope (95% CI); O:E, observed-to-expected ratio (95% CI).

Figure 2 presents the calibration plots for risk prediction of repeat self-harm within 1 and 6 months, including corresponding intercepts, slopes and O:E ratios. In the geographic external validation, there was a tendency for the model to underestimate risk for both prediction horizons (O:E for 1-month 1.22 (95% CI=1.13 to 1.32); for 6 months 1.15 (95% CI=1.09 to 1.21)). For 6 months, the calibration slope was higher than 1, mainly due to underestimation of risk in higher-risk persons. An exploratory analysis by region to further investigate possible sources of poorer calibration in the external sample showed some evidence of variation in performance by geographical region, with the Malmö and Västmanland regions showing the largest deviations, and some evidence of increased prevalence of risk factors such as history of self-harm in Västmanland (online supplemental tables 11 and 12). There was greater underestimation of risk in the temporal external validation sample, with a O:E of 1.54 (95% CI=1.47 to 1.62) at 1 month and 1.26 (95% CI=1.22 to 1.30) at 6 months. Meanwhile, the calibration slope was close to one at both prediction horizons, indicating that the underestimation of risk was relatively uniform across predicted risks. This was similar when using the composite outcome in the temporal validation sample (online supplemental figure 5).

## Discussion

In a sample of 53 172 people with self-harm presentations to hospital and other secondary healthcare services in the total Swedish population, we developed a risk assessment model for repeat self-harm within 1 month and 6 months of an index self-harm episode. The prevalence of repeat self-harm was 4% within 1 month and 9% within 6 months. In external validations (geographic and temporal), we found c-statistics of 0.66–0.68 for the 1-month model and 0.70–0.72 for the 6-month model. Using a 10% risk cut-off for self-harm within 6 months, we found that more than 50% of those went on to engage in self-harm (sensitivity)—and 76%–81% of those who did not engage in self-harm (specificity)—were correctly classified.

Our study improves on prior risk assessment and prediction tools in several ways. First, we had access to a large, nationally representative, sample of self-harm events, allowing sufficient power to predict repeat self-harm within both 1 and 6 months of self-harm. Short prediction horizons may give more clinically actionable information.^24^ By contrast, prior risk assessment models often derive from smaller and localised samples.^3 4^ Second, the one previous model developed in a sample of a similar size used a random split for development and validation samples,^5^ likely overestimating model performance. Our geographically and temporally based validation samples are preferred as more realistic assessments of model performance.^25^ Third, two previous investigations^4 18^ included several events per individual but did not account for the clustering of events within persons. For individuals with more than one recorded self-harm event in our data, we randomly selected an event to be defined as the index self-harm. This aligns with the intended use of the model and means that we can use information on past self-harm when predicting risk for new individuals.^9^

Importantly, no prior prediction models for repeat self-harm report calibration. Calibration measures how closely predicted probabilities match actual probabilities of an outcome—for a model to be clinically useful, it needs good calibration as well as discrimination.^26^ In OxSET, calibration was fair in geographic validation, with an O:E ratio close to 1. There was some underestimation of the predicted risk in higher risk persons, though this was limited to a few regions. In the temporal validation, there was underestimation of risk across predicted probabilities. Repeat self-harm was more common in temporal validation, possibly due to the greater prevalence of several markers of a high-risk population, including relatively more drug users and individuals with lifetime self-harm. The rate of self-harm has increased over the last decade in Sweden and other countries,^27^ and our prediction model may require recalibration for samples with higher event rates.

We have previously developed a tool for predicting suicide in individuals presenting with an index self-harm event, OxSATS,^9^ which showed a higher c-statistic (0.77). Risk factors in OxSATS were mostly overlapping with OxSET, but with some important differences. Female sex was a risk factor for repeat self-harm, in contrast to male sex in OxSATS. We also found evidence for an age-sex interaction in the current model unlike in OxSATS, alongside several additional predictors, including treatment features and family history of psychiatric disorders. While current or lifetime alcohol use disorder had no association with suicide in OxSATS, it was associated with increased risk of repeat self-harm in the current model. This could reflect a different underlying causal pathway, or that non-fatal self-harm is more common.

### Clinical implications

Well-performing risk assessment models may assist clinical decision-making rather than replace it. Unassisted clinician risk classification of repeated self-harm has been found to suffer from low performance, with an estimated sensitivity of 31% (95% CI=18% to 50%) and a specificity of 85% (75% to 92%) in a systematic review.^28^ For self-harm over 6 months at the 10% risk cut-off, the sensitivity of 52% in the geographic validation and 57% in the temporal validation suggests that our model identifies a higher proportion of those who repeat self-harm. Meanwhile, the classification metrics at 1 month with a 10% cut-off (sensitivity of 26%–29% and specificity of 91%–93%) suggests that, at this time horizon and cut-off, our model identifies a higher proportion of individuals who do not repeat self-harm. The most appropriate risk cut-off and prediction horizon will depend on local clinical context, including balancing true and false positives and negatives with available treatment options.^29^ The use of OxSET can provide consistency to risk assessment, where current practice appears to use many different unvalidated tools.^15^

An alternative to a binary cut-off (elevated/low) is to provide continuous probability risk scores to clinicians, allowing for more personalised assessment than a binary categorization.^26^ In clinical use, these probability scores will likely need one or more thresholds, which may differ by clinical setting. Such thresholds can be determined in expert clinical guidelines or by local services. Either alternative requires evidence of good calibration at relevant thresholds. OxSET could be employed as a screening or triaging instrument so that resources are focused on those at higher risk of repeat self-harm; alternatively, as every patient receives a probability score, the tool would also support clinical guidelines recommending safety planning and psychosocial assessments in everyone who self-harms. How you prioritise needs in resource-limited clinical settings will necessarily consider risk, and OxSET anchors such risk assessments in evidence. However, ultimately, any tool’s utility relies on availability of effective and scalable interventions to address the predicted risk.^9^

### Limitations

Some limitations should be noted. First, we did not have information on predictors likely to fluctuate over short time frames (eg, measures of suicidal ideation or mood symptoms).^24^ Second, a potential limitation to the interpretation of the risk score is the competing risk of suicide death.^30^ However, suicide was very rare—there were only 267 (0.7%) suicide deaths within 6 months. We employed an additional temporal external validation using a composite outcome of repeat non-fatal self-harm and death by suicide, and model performance was essentially unchanged. To capture the distinct risk factors for death by suicide as opposed to repeat self-harm, clinicians may consider using the current tool in conjunction with OxSATS. Third, while the model benefitted from a large and nationally representative data linkage with high validity of the routinely measured risk factors,^11^ further work is necessary to determine whether our model performs well in other contexts, in particular given evidence that performance varied by region. The discrimination performance of the model in the temporal external validation supports the use of the model in other contexts, although recalibration should be considered for higher-risk populations. Finally, the model was developed for self-harm presentations to secondary services, and is not validated for use in primary care or non-healthcare settings.

## Conclusion

In emergency departments, a scalable risk assessment tool for self-harm presentations may assist clinical decision-making, reduce the inconsistency and variability in how risk is assessed, and promote safety planning, further psychosocial evaluation and more efficient resource allocation. How such tools can link with community mental health and other services needs further work.

## Supplementary material

10.1136/bmjment-2024-301180 online supplemental file 1

## Data Availability

Data may be obtained from a third party and are not publicly available.
