# Supplementary material for: Risk of repeat self-harm among individuals presenting to healthcare services: development and validation of a clinical risk assessment model (OxSET)
Source: BMJ Ment Health. 2024 Oct 15;27(1):e301180. doi: 10.1136/bmjment-2024-301180 (PMC11481133; doi:10.1136/bmjment-2024-301180)
Supplement: online supplemental file 1 [file bmjment-27-1-s001.pdf]

Supplementary materials

Risk of repeat self-harm among individuals presenting to healthcare services: development and validation of a clinical risk assessment tool (OxSET)

Contents

Supplementary Text.......... 2

Supplementary Table 1: Variable definitions.......... 3

Supplementary Table 2: Number of individuals presenting with a repeat self-harm event within differing periods of follow-up 6

Supplementary Table 3: Individuals presenting with a repeat self-harm event within the regions forming the development and geographic validation samples 7

Supplementary Table 4: Participant characteristics in development and geographic validation sample.......... 8

Supplementary Table 5: Parameters (and their 95% confidence intervals) in the final multivariable model.......... 11

Supplementary Table 6: Unadjusted associations between candidate predictors and outcome in the development sample.......... 12

Supplementary Table 7: Internal discrimination performance of the final model. 14

Supplementary Table 8: Classification measures (and 95% confidence intervals) for the final model in the development sample 15

Supplementary Table 9: Discrimination performance in external validation samples 15

Supplementary Table 10: Classification measures (and 95% confidence intervals) for the final model evaluated in the external validation samples.......... 16

Supplementary Table 11: Observed to expected (O:E) outcome ratios in the regions included in the external validation sample.......... 17

Supplementary Table 12: Prediction factor distribution in the external validation regions with largest deviation from the overall observed:expected ratio 18

Supplementary Figure 1: Illustration of age:sex interaction effect in the final risk prediction model. 20

Supplementary Figure 2: ROC curves in the development sample.......... 21

Supplementary Figure 3: Internal calibration plots.......... 22

Supplementary Figure 4: ROC curve and AUC (95%CI) for the final model when used to predict risk of repeat self-harm at 1 and 6 months in the external temporal validation sample, using the composite outcome... 23

Supplementary Figure 5: Calibration plots for the final multivariable model used to predict repeat self-harm in the external temporal validation sample, using the composite outcome. 24

Supplementary Text.

Following previous studies, we used four geographical regions stratified by population density: (1) major urban centres, (2) counties with major urban centres removes, (3) counties with small population density, and (4) counties with medium population density [27, 28]. To form an 'external validation sample', regions were randomly selected in such a way that the number of suicide outcome events (the primary outcome in the sister study) exceeded 100 [10]. The remaining regions were used for model development.

Supplementary Table 1: Variable definitions

**Outcome definition**	
Repeat self-harm	A subsequent self-harm event within<br>(a) 1 month after the index self-harm event, and<br>(b) 6 months after the index self-harm event<br>ICD-10: X60-X84 (intentional self-harm) or Y10-Y33 (events of undetermined intent), but excluding Y34 (method of self-harm not recorded)
**General demographics**	
Age*	In years, at time of index date; individuals less than 10 years excluded
Sex*	Coded as male or female
**Substance misuse**	
Current or lifetime alcohol use disorder, excluding acute intoxication*	At any time at or before index date<br>ICD-10: F10.1-F10.6 (acute intoxication (F10.0) excluded)<br>ICD-9: 291, 303.9, 305.0 (nondependent alcohol<br>ICD-8: 291, 303.0, 303.1, 303.2, 303.9
Current or lifetime drug use disorder*	At any time at or before index date<br>ICD-10: F11-F16, F18-19 (subdivisions .1-.7)<br>ICD-9: 292, 304, 305.2-305.9<br>ICD-8: 304
Alcohol intoxication at index	At time of index date<br>ICD-10: F10.0
**Living situation**	Obtained from LISA register (adults defined as aged 16+ years)
Living with adult	Living with at least one other individual aged 16+ years. All individuals under 16 years old with missing living situation were reclassified as 'living with adult'
Living with children	Living with at least one individual under 16 years old
**Treatment in the past three months**	Dispensed in the three months prior to index date
Any psychotropic medication	Any prescription of antidepressant, antipsychotic or mood stabiliser medication (defined as below)

Antidepressant treatment	ATC: N06A
Antipsychotic treatment	ATC: N05A, excluding N05AN01, N05AH02
Mood stabilizer treatment	ATC: N03AG01, N03AX09, N03AF01, N03AN01
**Physical health problems**	
New cancer diagnosis	An instance of a cancer diagnosis (ICD-10: C00-D48) occurring within 12 months before index date that is not a relapse (recoding) of a previous diagnosis made in the preceding 2 years
**History of self-harm**	
Any psychotropic medication overdose	Psychotropic medication overdose (ICD-10: X61 or Y11) as the method of harm of the index event
Cutting	Cutting (i.e. sharp object, ICD-10: X78 or Y28) as the method of harm of the index event
Hanging, strangulation or suffocation	Hanging, strangulation or suffocation (ICD-10: X70 or Y20) as the method of harm of the index event
Drowning	Drowning or submersion (ICD-10: X71 or Y21) as the method of harm of the index event
Lifetime history of self-harm prior to index*	Any instance of self-harm occurring prior to the index date<br>ICD-10: X60-X84, Y10-Y33<br>ICD-8/9: E950-E959 and E980-E987
History of self-harm in the 12 months prior to index*	Any instance of self-harm occurring within the 12 months before the index date, codes as in row above
Number of lifetime prior self-harm episodes	Binary indicator of the number of past self-harm events, including the index self-harm event, 1-2 vs 3 or more events
Overnight admission	Binary indicator of whether the index event resulted in an overnight hospital admission
Any self-harm occurring within one month prior to index date	Any instance of self-harm (ICD-10: X60-X84, Y10-Y33) occurring within one month before the index date
**Mental health in the past 12 months**	
Any psychiatric disorder except substance use disorders	ICD-10: F00-F09, F20-F99 (Mental and behavioural disorders, excluding those due to psychoactive substance use)
Serious psychiatric disorder	ICD-10: F20-F29 (Schizophrenia, schizotypal and delusional disorders), F30-F31 (Mood [affective] disorders)

**Criminal/violence/legal issues**	Obtained from National Crime Register; individuals younger than 15 (the age of criminal responsibility) are excluded from the definitions below
Lifetime criminal record for any crime	Conviction for any crime before the index date
Criminal record for any crime in past 12 months	Conviction for any crime within 12 months before the index date
Lifetime arrest history for any crime	Arrest for any crime before the index date
Arrest history for any crime in past 12 months	Arrest for any crime within 12 months before the index date
Lifetime criminal record for violent crime	Conviction for any of the following before the index date: attempted/completed/aggravated forms of homicide, manslaughter, unlawful threats, harassment, robbery, arson, assault, assault on an official, kidnapping, stalking, coercion, all sexual offences
Criminal record for violent crime in past 12 months	Conviction for any violent crime, as listed above, within 12 months before the index date
Lifetime arrest history for violent crime	Arrest for any violent crime, as listed above before the index date
Arrest history for violent crime in the past 12 months	Arrest for any violent crime, as listed above, within 12 months before the index date
**Family history**	
Family history of suicide	Death from suicide (ICD-10: X60-X84, Y10-Y33) of any parent, sibling or child before the index date
Family history of any psychiatric disorder	Diagnosis of psychiatric disorder (ICD-10: F00-09, F20-F99) of any parent, sibling or child before the index date

Note: all variables except age were coded as binary variables. Those marked with an asterisk (*) are core risk factors that were included in the final model irrespective of statistical significance.

ATC: Anatomical Therapeutic Chemical Classification System

ICD: International Statistical Classification of Diseases and Related Health Problems

LISA: Longitudinal integrated database for health insurance and labour market studies, https://www.scb.se/lisa-en

Supplementary Table 2: Number of individuals presenting with a repeat self-harm event within differing periods of follow-up

		External validation		
	**Development sample N=37,523**	**Geographic N=15,649**	**Temporal N=25,036**	
**Outcome**	**Repeat non-fatal self-harm**	**Repeat non-fatal self-harm**	**Repeat non-fatal self-harm**	**Composite of repeat non-fatal self-harm and suicide**
**Within 1 month**	1,259 (3.36%)	648 (4.14%)	1,591 (6.35%)	1,702 (6.80%)
**Within 6 months**	2,820 (7.52%)	1,373 (8.77%)	2,886 (11.53%)	3,101 (12.39%)

Supplementary Table 3: Individuals presenting with a repeat self-harm event within the regions forming the development and geographic validation samples

Group 1	Group 2	Group 3	Group 4
Major urban centres	Counties with major urban centres removed	Counties with small population (5 to 28 suicide events)	Counties with medium population (24 to 59 suicide events)
Development sample			
1480 Gothenburg<br>[65, 2%] {189, 7%}*	1 Stockholm County Other<br>[201, 3%] {496, 7%}	7 Kronoberg<br>[40, 4%] {80, 9%}	3 Uppsala<br>[86, 4%] {190, 9%}
1801 Stockholm City North<br>[64, 3%] {147, 7%}	12 Skåne Other<br>[185, 4%] {390, 8%}	9 Gotland<br>[16, 4%] {29, 8%}	4 Södermanland<br>[61, 3%] {148, 7%}
1802 Stockholm City South<br>[86, 3%] {214, 7%}		10 Blekinge<br>[26, 3%] {46, 5%}	6 Jönköping<br>[120, 6%] {213, 11%}
			8 Kalmar<br>[60, 5%] {119, 9%}
			18 Örebro<br>[33, 2%] {80, 6%}
			20 Dalarna<br>[43, 3%] {84, 6%}
			21 Gävleborg<br>[45, 3%] {101, 7%}
			22 Västernorrland<br>[32, 3%] {79, 8%}
			24 Västerbotten<br>[36, 3%] {89, 7%}
			25 Norrbotten<br>[60, 4%] {126, 8%}
External validation sample			
1280 Malmö<br>[77, 5%] {134, 9%}	14 Västra Götaland Other<br>[175, 3%] {418, 7%}	23 Jämtland<br>[12, 2%] {27, 5%}	5 Östergötland<br>[131, 5%] {274, 10%}
			13 Halland<br>[66, 4%] {141, 9%}
			17 Värmland<br>[64, 4%] {152, 9%}
			19 Västmanland<br>[123, 6%] {227, 11%}

* [Within 1 month] {Within 6 months}

Supplementary Table 4: Participant characteristics in development and geographic validation sample

	Development sample N=37,523		Geographic validation sample N=15,649	
**General demographics**	**N**	**%**	**N**	**%**
Age (years)[1]		Median=32, IQR=21-49		Median=33, IQR=21-49
Individuals under 16 years	2,224	5.93	864	5.52
Sex, Female[1]	20,561	54.80	8,685	55.50
**Substance misuse**				
Current or alcohol use disorder[1]	7,257	19.34	3,025	19.33
Current or lifetime drug use disorder[1]	8,384	22.34	3,672	23.46
Alcohol intoxication at index	1,416	3.77	815	5.21
**Living situation[2]**				
Living with other adult	14,151	37.71	5,697	36.40
Living with children	15,493	41.29	6,494	41.50
**Treatment in the past three months**				
Any psychotropic medication	20,888	55.67	9,537	60.94
Antidepressant treatment	12,527	33.38	6,009	38.40
Antipsychotic treatment	4,259	11.35	1,834	11.72
Mood stabilizer treatment	677	1.80	310	1.98
**Physical health problems**				
New cancer diagnosis	411	1.1	149	1.0
**History of self-harm**				
Method of index self-harm event[3]				
- Any psychotropic medication overdose	1,360	3.62	539	3.44
- Cutting	4,925	13.13	1,614	10.31
- Hanging, strangulation or suffocation	308	0.82	150	0.96
- Drowning	51	0.14	23	0.15
Lifetime history of self-harm prior to index[1]	11,277	30.05	5,372	34.33

	Development sample N=37,523		Geographic validation sample N=15,649	
History of self-harm in the 12 months prior to index[1]	4,281	11.41	1,994	12.74
Number of lifetime prior self-harm episodes				
1-2 episodes	31,740	84.59	12,879	82.30
3+ episodes	5,783	15.41	2,770	17.70
Overnight admission	16,991	45.28	7,531	48.12
Time between episodes <= 1 month	1,935	5.16	838	5.36
**Mental health in the past 12 months**				
Any psychiatric disorder (except substance use disorders)	16,472	43.90	7,281	46.53
Serious psychiatric disorder	3,006	8.01	1,305	8.34
**Criminal/violence/legal issues**				
Lifetime criminal record for any crime	13,451	35.85	5,974	38.17
Criminal record for any crime in past 12 months	3,816	10.17	1,809	11.56
Lifetime arrest history for any crime	15,068	40.16	6,506	41.51
Arrest history for any crime in past 12 months	6,416	17.10	2,818	18.01
Lifetime criminal record for violent crime	5,729	15.27	2,576	16.46
Criminal record for violent crime in past 12 months	897	2.39	416	2.66
Lifetime arrest history for violent crime	5,402	14.40	2,266	14.48
Arrest history for violent crime in the past 12 months	2,141	5.71	872	5.57
**Family history**				
Family history of suicide	1,118	2.98	530	3.39
Family history of any psychiatric disorder	15,112	40.27	6,503	41.56

[1] Core factor, kept in the final model independently of its statistical significance or predictive strength

[2] Living situation information was missing for 641 (2%) individuals in the development sample and 199 (1%) in the geographic validation sample. Individuals under 16 years old with missing living situation were reclassified as 'living with other adult' (2,049 (92%) of the under 16s in the development sample; 815 (94%) in the geographic validation sample). Of the 641 individuals with missing living situation in the development sample, 22 (3%) presented with a repeat

self-harm within 1 month, 18 (3%) between 1 and 6 months, and 23 (4%) more than 6 months since index. In the geographic validation sample the corresponding numbers are 6 (3%), 7 (4%), and 17 (9%).

[3] It was possible for an individual's index self-harm event to be coded under more than one of the methods listed. The majority of individuals not included in any of the listed categories had either non-psychotropic medicine overdose or an unspecified method of index self-harm.

Values are all numbers and percentages and 95% confidence intervals, except for age for which mean and standard deviation (SD) are reported.

Supplementary Table 5: Parameters (and their 95% confidence intervals) in the final multivariable model

	Parameter (β)	95% confidence interval	
**General demographics**			
Age in decades	-0.08	-0.11	-0.05
Female sex	0.50	0.34	0.65
Interaction of age (in decades) and sex	-0.09	-0.12	-0.05
**Substance misuse**			
Current or lifetime alcohol use disorder	0.24	0.17	0.31
Current or lifetime drug use disorder	0.37	0.30	0.44
**Living situation**			
Living with other adult	-0.08	-0.15	-0.02
**Treatment in the past 3 months**			
Any psychotropic medication	0.39	0.30	0.48
Antidepressant treatment	0.16	0.09	0.23
Antipsychotic treatment	0.18	0.10	0.25
Mood stabilizer treatment	0.17	0.02	0.32
**History of self-harm**			
Method of index self-harm event			
Cutting	0.27	0.19	0.35
Hanging, strangulation or suffocation	0.49	0.24	0.74
Lifetime history of self-harm prior to index	0.20	0.11	0.29
History of self-harm in the 12 months prior to index	0.55	0.46	0.64
Number of lifetime prior self-harm episodes, 3+ episodes	0.53	0.45	0.62
Time between episodes <= 1 month	0.11	0.01	0.21
**Mental health in the past 12 months**			
Any psychiatric disorder (except substance use disorders)	0.37	0.30	0.45
**Family history**			
Family history of any psychiatric disorder	0.09	0.03	0.15
**Other parameters in the model**			
Intercept	-4.43	-4.58	-4.29
Shape parameter	0.48	0.47	0.50

The formula to calculate the risk of repeat self-harm within *t* months following an emergency treatment for self-harm is:

$$\Pr(repeat\ self-harm\ within\ t\ months) = 1 - \exp(-\exp(linpred_{sh})\ t^{0.48}),$$

where $linpred_{sh} = -4.43 + \sum \beta * Risk\ Factor$ and $t = 1$ or 6.

The baseline survivor function is given by $S_0(t) = \exp(-0.01 * t^{0.48})$. At 1 and 6 months this is $S_0(1) = 0.9882$ and $S_0(6) = 0.97$, respectively.

Only age is continuous, the rest are binary factors coded as 1 if present and 0 otherwise.

Supplementary Table 6: Unadjusted associations between candidate predictors and outcome in the development sample

	Hazard Ratio	95% CI		p-value	Weibull shape parameter	95% CI	
**General demographics**							
Age in decades (centred around its mean of 37 years)	0.93	0.91	0.94	<0.001	0.4608	0.4481	0.4738
Female sex	1.46	1.37	1.54	<0.001	0.4611	0.4484	0.4741
**Substance misuse**							
Current or lifetime alcohol use disorder	1.97	1.85	2.09	<0.001	0.4621	0.4494	0.4751
Current or lifetime drug use disorder	2.59	2.44	2.74	<0.001	0.4643	0.4516	0.4774
Alcohol intoxication at index	1.15	1.00	1.32	0.050	0.4606	0.4479	0.4736
**Living situation**							
Living with other adult	0.68	0.64	0.73	<0.001	0.4625	0.4497	0.4756
Living with children	0.90	0.85	0.96	0.001	0.4620	0.4492	0.4751
**Treatment in the past three months**							
Any psychotropic medication	2.68	2.51	2.87	<0.001	0.4637	0.4510	0.4768
Antidepressant treatment	2.22	2.10	2.35	<0.001	0.4634	0.4507	0.4764
Antipsychotic treatment	2.59	2.42	2.77	<0.001	0.4636	0.4509	0.4767
Mood stabilizer treatment	2.39	2.06	2.77	<0.001	0.4610	0.4483	0.4741
**Physical health problems**							
New cancer diagnosis	1.24	0.97	1.58	0.090	0.4606	0.4479	0.4736
**History of self-harm**							
Method of self-harm at index							
Any psychotropic medication overdose	1.39	1.22	1.59	<0.001	0.4606	0.4479	0.4736
Cutting as a method of harm	1.12	1.03	1.21	0.008	0.4606	0.4479	0.4736
Hanging, strangulation or suffocation	1.68	1.31	2.16	<0.001	0.4606	0.4479	0.4736
Drowning	0.94	0.42	2.09	0.873	0.4606	0.4479	0.4736
Lifetime history of self-harm prior to index	3.41	3.22	3.61	<0.001	0.4671	0.4543	0.4802
History of self-harm in the 12 months prior to index	4.18	3.93	4.44	<0.001	0.4693	0.4566	0.4825

Number of lifetime prior self-harm episodes, 3+ episodes	4.13	3.89	4.38	<0.001	0.4697	0.4569	0.4828
Time between lifetime episodes <=1 month	3.17	2.92	3.46	<0.001	0.4634	0.4507	0.4765
Overnight admission	1.12	1.06	1.19	<0.001	0.4606	0.4479	0.4736
**Mental health in the past 12 months**							
Any psychiatric disorder except substance use disorders	3.08	2.90	3.28	<0.001	0.4657	0.4529	0.4788
Serious psychiatric disorder	2.06	1.90	2.24	<0.001	0.4617	0.4490	0.4748
**Criminal/violence/legal issues**							
Lifetime criminal record for any crime	1.31	1.24	1.39	<0.001	0.4608	0.4481	0.4738
Criminal record for any crime in past 12 months	1.38	1.27	1.50	<0.001	0.4607	0.4480	0.4738
Lifetime arrest history for any crime	1.46	1.38	1.55	<0.001	0.4611	0.4484	0.4741
Arrest history for any crime in past 12 months	1.49	1.39	1.59	<0.001	0.4610	0.4483	0.4740
Lifetime criminal record for violent crime	1.40	1.30	1.50	<0.001	0.4608	0.4481	0.4738
Criminal record for violent crime in past 12 months	1.40	1.19	1.64	<0.001	0.4606	0.4479	0.4736
Lifetime arrest history for violent crime	1.59	1.48	1.70	<0.001	0.4612	0.4485	0.4742
Arrest history for violent crime in the past 12 months	1.49	1.34	1.66	<0.001	0.4607	0.4481	0.4738
**Family history**							
Family history of suicide	1.27	1.10	1.48	0.002	0.4606	0.4479	0.4736
Family history of any psychiatric disorder	1.38	1.30	1.46	<0.001	0.4611	0.4484	0.4741

Supplementary Table 7: Internal discrimination performance of the final model.

	Apparent			Adjusted for optimism		
	Overall	1 month	6 months	Overall	1 month	6 months
	Statistic (95% CI)	Statistic (95% CI)	Statistic (95% CI)	Statistic (95% CI)	Statistic (95% CI)	Statistic (95% CI)
**Final model**						
**Harrell's c**	0.73 (0.73, 0.74)	0.69 (0.68, 0.71)	0.73 (0.72, 0.74)	0.73 (0.72, 0.74)	0.69 (0.67, 0.71)	0.73 (0.72, 0.74)
**Somers' D**	0.47 (0.45, 0.48)	0.39 (0.35, 0.42)	0.46 (0.44, 0.48)	0.47 (0.45, 0.48)	0.39 (0.34, 0.43)	0.46 (0.44, 0.48)
**Crime model**						
**Harrell's c**	0.73 (0.73, 0.74)	0.69 (0.68, 0.71)	0.73 (0.72, 0.74)	0.73 (0.72, 0.74)	0.69 (0.67, 0.71)	0.73 (0.72, 0.74)
**Somers' D**	0.47 (0.45, 0.48)	0.39 (0.36, 0.42)	0.46 (0.44, 0.48)	0.47 (0.45, 0.49)	0.39 (0.35, 0.43)	0.46 (0.44, 0.49)

Supplementary Table 8: Classification measures (and 95% confidence intervals) for the final model in the development sample

	Risk at 1 month Prevalence = 3.4% (3.2, 3.5)			Risk at 6 months Prevalence =7.5% (7.3, 7.8)		
Thresholds	10%	20%	25%	10%	20%	25%
Sensitivity	27.7% (25.3, 30.3)	7.6% (6.2, 9.2)	4.1% (3.1, 5.4)	51.5% (49.6, 53.3)	30.2% (28.5, 31.9)	23.1% (21.6, 24.7)
Specificity	93.4% (93.1, 93.6)	97.8% (97.6, 97.9)	98.2% (98.0, 98.3)	82% (81.6, 82.4)	93.3% (93.0, 93.6)	95.3% (95.1, 95.5)
PPV	12.7% (11.5, 14.0)	10.5% (8.6, 12.7)	7.3% (5.5, 9.5)	18.8% (18.0, 19.7)	26.8% (25.2, 28.4)	28.6% (26.8, 30.5)
NPV	97.4% (97.2, 97.5)	96.8% (96.6, 97)	96.7% (96.5, 96.9)	95.4% (95.2, 95.6)	94.3% (94.0, 94.5)	93.8% (93.6, 94.1)

PPV = Positive predictive value; NPV = Negative predictive value

Supplementary Table 9: Discrimination performance in external validation samples

	**Geographic validation**		**Temporal validation**			
	**Repeat non-fatal self-harm**		**Repeat non-fatal self-harm**		**Composite of repeat non-fatal self-harm and suicide**	
	**1 month**	**6 months**	**1 month**	**6 months**	**1 month**	**6 months**
	**Statistic (95% CI)**	**Statistic (95% CI)**	**Statistic (95% CI)**	**Statistic (95% CI)**	**Statistic (95% CI)**	**Statistic (95% CI)**
**Harrell's c**	0.68 (0.65, 0.70)	0.72 (0.70, 0.73)	0.66 (0.64, 0.67)	0.70 (0.69, 0.71)	0.65 (0.63, 0.66)	0.70 (0.69, 0.71)
**Somers' D**	0.35 (0.31, 0.40)	0.43 (0.40, 0.46)	0.31 (0.28, 0.34)	0.41 (0.39, 0.43)	0.29 (0.27, 0.32)	0.39 (0.37,0.41)

Supplementary Table 10: Classification measures (and 95% confidence intervals) for the final model evaluated in the external validation samples

**Geographic validation**	**Repeat non-fatal self-harm**	**Risk at 1 month Prevalence = 4.1% (95% CI: 3.8, 4.5)**			**Risk at 6 months Prevalence = 8.8% (95% CI: 8.3, 9.2)**		
		**10%**	**20%**	**25%**	**10%**	**20%**	**25%**
	Sensitivity	26.4% (23.0, 30.0)	6.3% (4.6, 8.5)	1.9% (1.0, 3.2)	51.5% (48.8, 54.2)	31.0% (28.5, 33.5)	22.3% (20.1, 24.6)
	Specificity	93.1% (92.7, 93.5)	98.1% (97.9, 98.3)	98.6% (98.4, 98.8)	80.7% (80.1, 81.4)	93.0% (92.6, 93.4)	95.3% (95.0, 95.7)
	PPV	14.2% (12.3, 16.3)	12.7% (9.3, 16.8)	5.4% (2.8, 9.3)	20.5% (19.1, 21.8)	29.9% (27.5, 32.4)	31.5% (28.6, 34.5)
	NPV	96.7% (96.4, 97.0)	96.0% (95.7, 96.3)	95.9% (95.6, 96.2)	94.5% (94.1, 94.9)	93.3% (92.9, 93.7)	92.7% (92.3, 93.1)
**Temporal validation**	**Repeat non-fatal self-harm**	**Risk at 1 month Prevalence = 6.4% (95% CI: 6.1, 6.7)**			**Risk at 6 months Prevalence = 12.0% (95% CI: 11.0, 11.9)**		
		**10%**	**20%**	**25%**	**10%**	**20%**	**25%**
	Sensitivity	28.6% (26.4, 30.9)	8.99% (7.6, 10.5)	3.14% (2.3, 4.1)	56.9% (55.1, 58.7)	35.0% (33.2, 36.7)	28.0% (26.4, 29.7)
	Specificity	91.3% (91.0, 91.7)	98.6% (98.4, 98.7)	99.5% (99.4, 99.6)	76.0% (75.5, 76.6)	91.3% (90.9, 91.7)	94.5% (94.2, 94.8)
	PPV	18.3% (16.8, 19.9)	29.9% (25.8, 34.2)	31.4% (24.3, 39.3)	23.6% (22.6, 24.6)	34.4% (32.6, 36.1)	40.1% (37.9, 42.2)
	NPV	95.0% (94.7, 95.2)	94.1% (93.8, 94.4)	93.8% (93.5, 94.1)	93.1% (92.7, 93.5)	91.5% (91.1, 91.9)	91.0% (90.6, 91.3)
	**Composite outcome**	**Risk at 1 month prevalence = 6.8% (95% CI: 6.5, 7.1)**			**Risk at 6 months Prevalence = 12.0% (95% CI: 12.0, 12.8)**		
		**10%**	**20%**	**25%**	**10%**	**20%**	**25%**
	Sensitivity	27.4% (25.3, 29.6)	8.52% (7.24, 9.95)	2.94% (2.19, 3.85)	55.4% (53.6, 57.2)	33.5% (31.8, 35.2)	26.8% (25.2, 28.4)
	Specificity	91.3% (91.0, 91.7)	98.6% (98.4, 98.7)	99.5% (99.4, 99.6)	76.2% (75.6, 76.7)	91.4% (91.0, 91.7)	94.6% (94.3, 94.9)
	PPV	18.8% (17.2, 20.4)	30.3% (26.2, 34.6)	31.4% (24.3, 39.3)	24.7% (23.7, 25.8)	35.4% (33.7, 37.1)	41.2% (39.0, 43.4)
	NPV	94.5% (94.2, 94.8)	93.7% (93.3, 94.0)	93.4% (93.0, 93.7)	92.4% (92.0, 92.7)	90.7% (90.3, 91.1)	90.1% (89.7, 90.5)

Notes: PPV = Positive predictive value; NPV = Negative predictive value

Supplementary Table 11: Observed to expected (O:E) outcome ratios in the regions included in the external validation sample

	1 month			6 months		
	N	O	O:E (95%CI)	N	O	O:E (95%CI)
Malmö	1,439	77	1.69 (1.36,2.10)	1,439	134	1.31 (1.11,1.53)
Västra Götaland Other	5,682	175	0.91 (0.79,1.06)	5,682	418	0.97 (0.88,1.06)
Jämtland	501	12	0.78 (0.44,1.36)	501	27	0.77 (0.53,1.11)
Östergötland	2,819	131	1.39 (1,17,1.64)	2,819	274	1.29 (1.15,1.44)
Halland	1,485	66	1.26 (1.00,1.60)	1,485	141	1.20 (1.02,1.40)
Värmland	1,696	64	1.13 (0.89,1.44)	1,696	152	1.19 (1.02,1.38)
Västmanland	2,027	123	1.65 (1.39,1.96)	2,027	227	1.35 (1.20,1.53)

Supplementary Table 12: Prediction factor distribution in the external validation regions with largest deviation from the overall observed:expected ratio

	Development sample		Östergötland		Västmanland		Jämtland		Malmö	
	N	%	N	%	N	%	N	%	N	%
Age (years)	Mean=37, SD=18		Mean=36, SD=18		Mean=37, SD=18		Mean=36, SD=19		Mean=37, SD=18	
Female sex	20,561	54.8	1,576	56	1,102	54	269	54	808	56
Current or lifetime alcohol use disorder	7,257	19.3	504	18	456	23	93	19	260	18
Current or lifetime drug use disorder	8,384	22.3	609	21.6	593	29.21	98	19.56	389	27.03
Living with other adult	14,151	37.7	1012	35.9	681	33.6	187	37.33	447	31.06
Any psychotropic medication	20,888	55.7	1649	58.5	1245	61.42	293	58.48	751	52.19
Antidepressant treatment	12,527	33.4	1078	38.24	744	36.7	183	36.53	430	29.88
Antipsychotic treatment	4,259	11.4	285	10.11	229	11.3	57	11.38	168	11.67
Mood stabilizer treatment	677	1.8	53	1.88	34	1.68	7	1.4	21	1.46
Cutting	4925	13.2	363	12.88	243	11.99	36	7.19	165	11.47
Hanging, strangulation or suffocation	308	0.8	33	1.17	28	1.38	3	0.6	11	0.76
Lifetime history of self-harm prior to index	11,277	30.1	981	34.8	811	40.01	169	33.73	456	31.69
History of self-harm in the 12 months prior to index	4,281	11.4	397	14.08	299	14.75	63	12.57	163	11.33

Number of prior episodes, 3+	5,783	15.4	486	17.24	434	21.41	85	16.97	242	16.82
Time between episodes <= 1 month	1,929	5.1	176	6.24	122	6.02	32	6.39	81	5.63
Any psychiatric disorder (except substance use disorders)	16,472	43.9	1215	43.1	976	48.15	207	41.32	594	41.28
Family history of any psychiatric disorder	15,112	40.3	1135	40.26	862	42.53	210	41.92	565	39.26

Supplementary Figure 1: Illustration of age:sex interaction effect in the final risk prediction model.

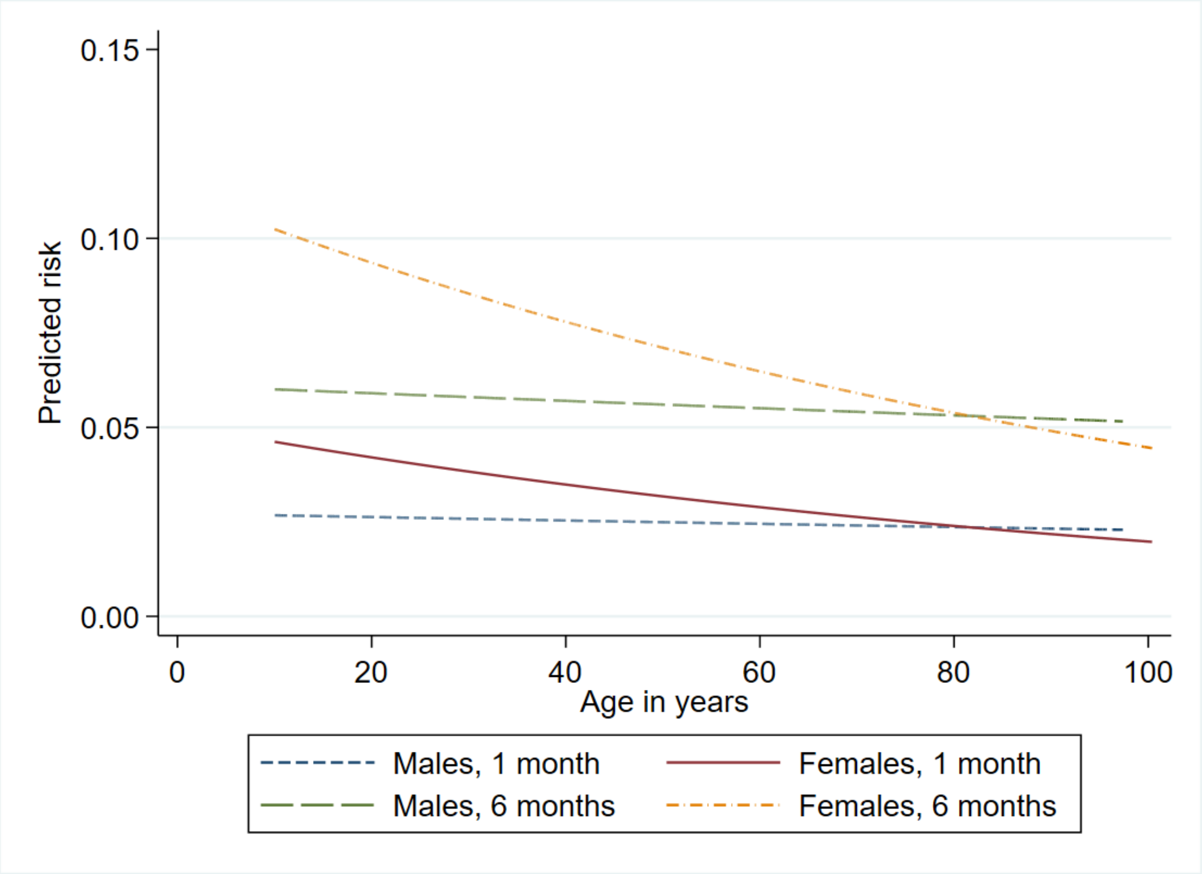

Predicted risk refers to risk within 1 and 6 months for males and females of different ages with all other factors fixed at zero (individual predicted risk is likely to increase in all groups if other risk factors are present).

Supplementary Figure 2: ROC curves in the development sample

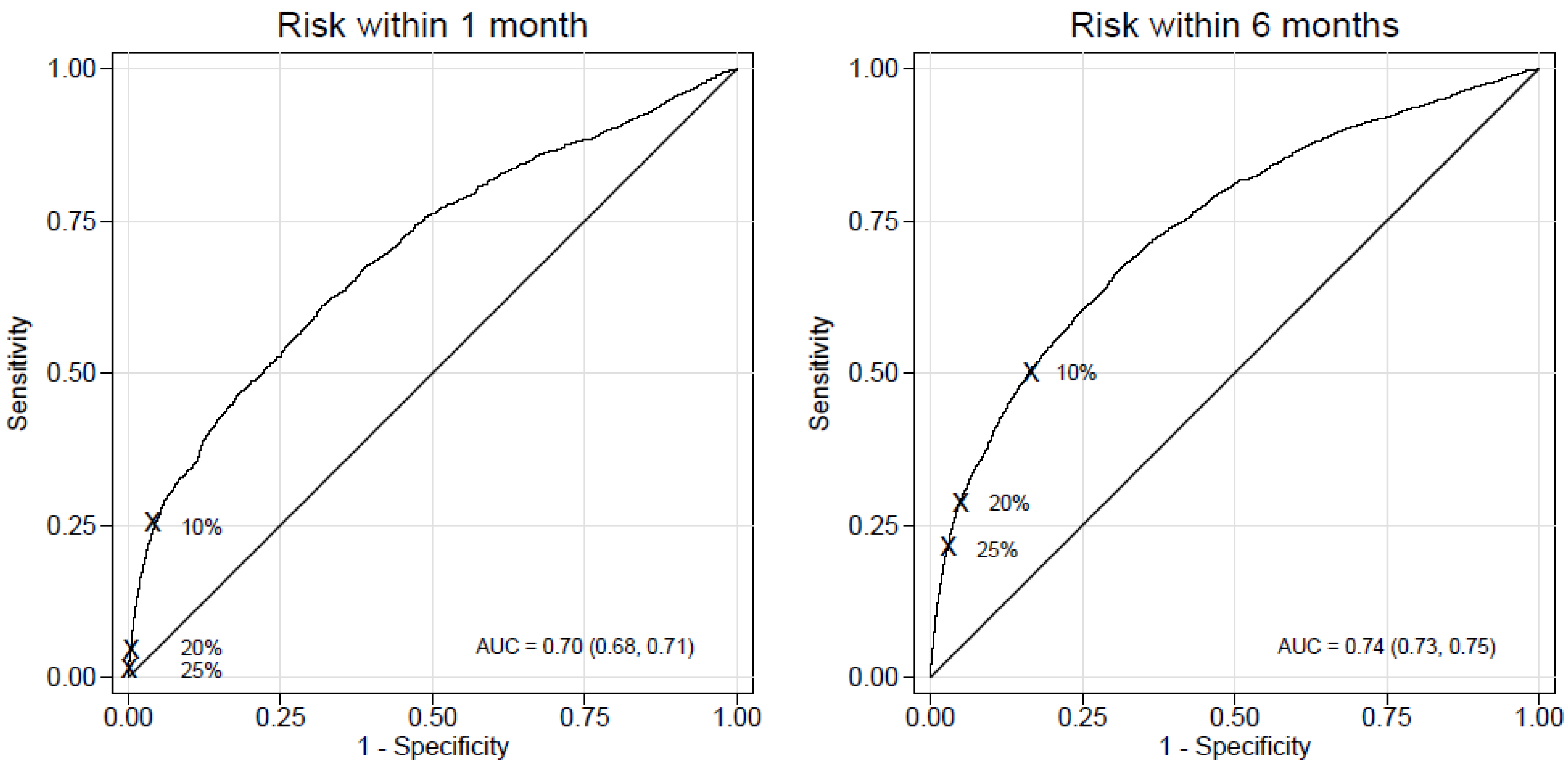

Values are Area under the ROC curve (AUC) and 95% confidence intervals; the points marked X correspond to different probability risk cut-points considered (10%, 20%, 25%).

Supplementary Figure 3: Internal calibration plots

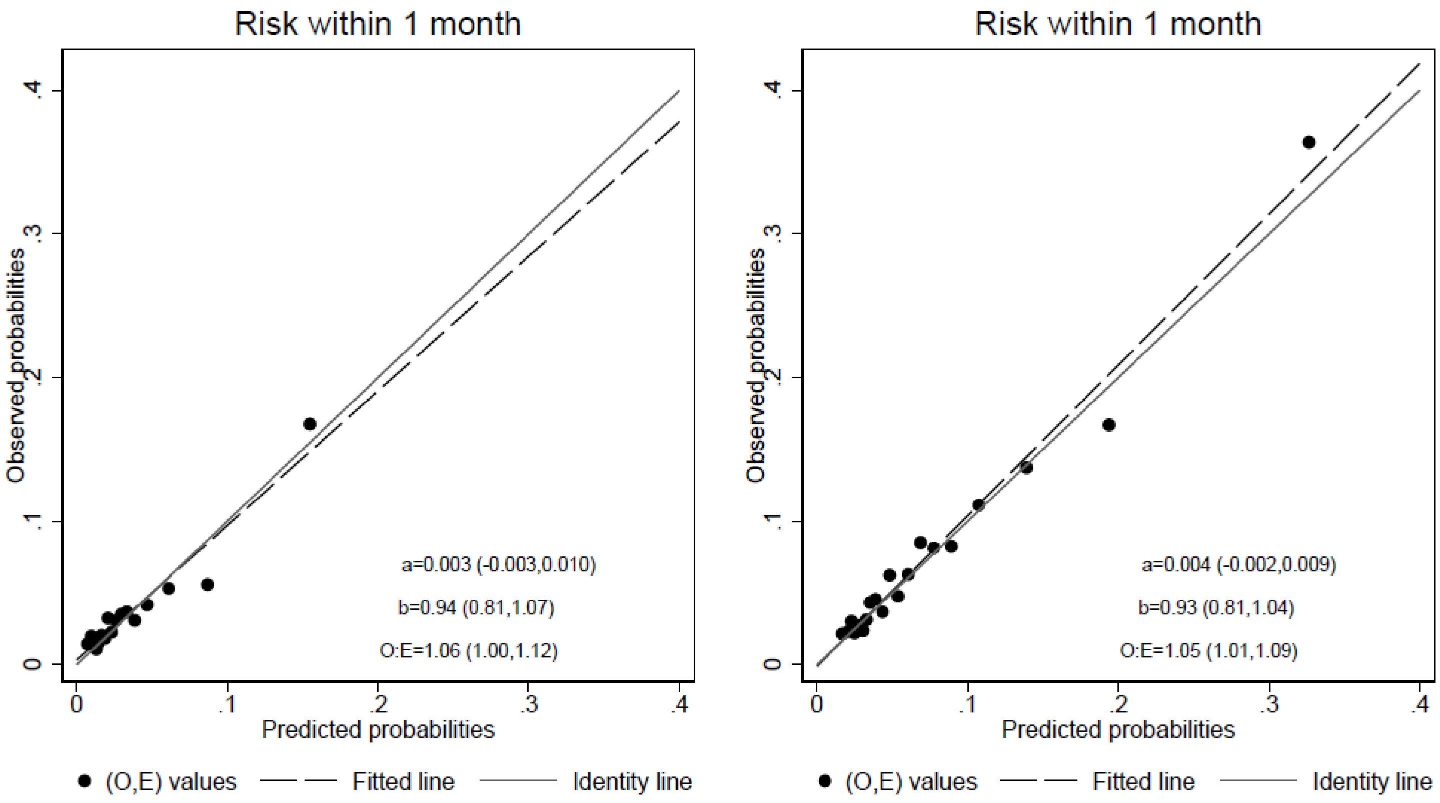

O:E = Observed to expected events ratio

Supplementary Figure 4: ROC curve and AUC (95%CI) for the final model when used to predict risk of repeat self-harm at 1 and 6 months in the external temporal validation sample, using the composite outcome.

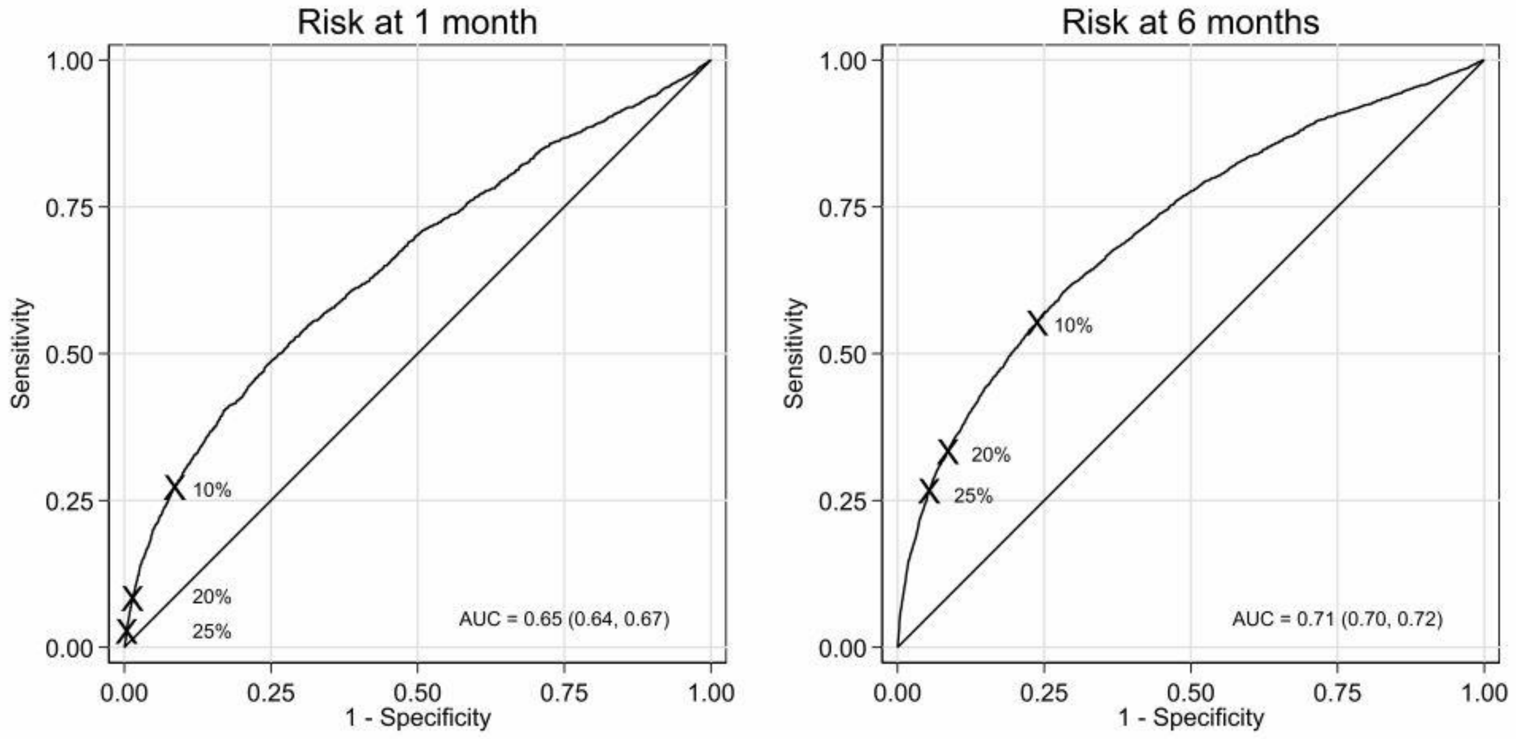

Values are Area under the ROC curve (AUC) and 95% confidence intervals; the points marked X correspond to different probability risk cut-points considered (10%, 20%, 25%).

Supplementary Figure 5: Calibration plots for the final multivariable model used to predict repeat self-harm in the external temporal validation sample, using the composite outcome.

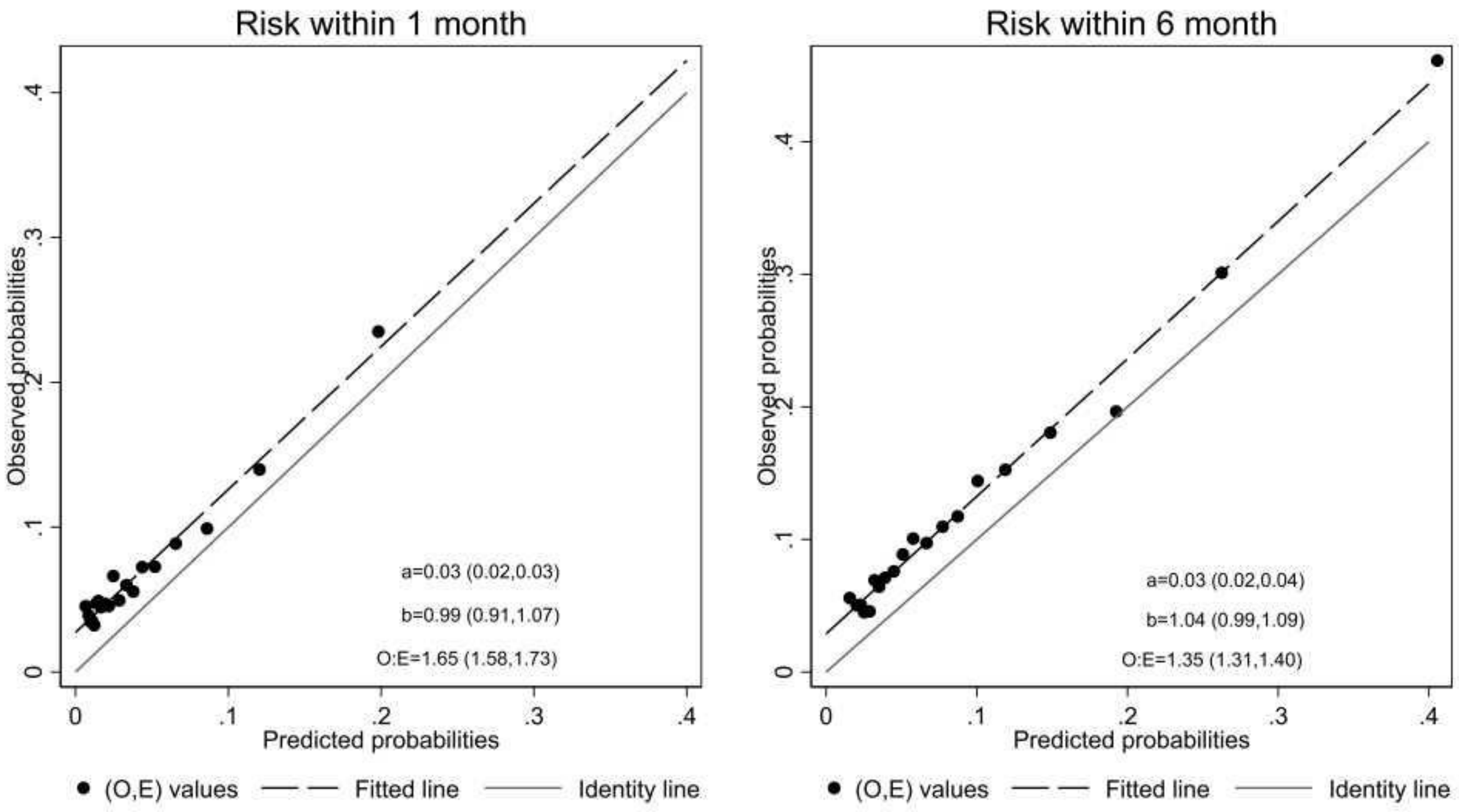

O:E = Observed to expected events ratio
